# NMR-Based Metabolomic Approach for Evaluation of the Harvesting Time and Cooking Characteristics of Different Cassava Genotypes

**DOI:** 10.3390/foods11111651

**Published:** 2022-06-03

**Authors:** Lorena Mara A. Silva, Elenilson G. Alves Filho, Robson M. Martins, Willyane J. D. J. Oliveira, Cristine S. Vidal, Luciana A. de Oliveira, Edy S. de Brito

**Affiliations:** 1 Embrapa Agroindústria Tropical, Rua Dra. Sara Mesquita, Pici 2270, Fortaleza 60511-110, Brazil; lorena.mara@embrapa.br; 2 Departamento de Engenharia de Alimentos, Campus do Pici, Universidade Federal do Ceará, Fortaleza 60440-900, Brazil; elenilson.godoy@yahoo.com.br; 3 Departamento de Química, Campus do Pici, Universidade Federal do Ceará, Fortaleza 60440-900, Brazil; robsonmm95@gmail.com (R.M.M.); willyane.jesca@gmail.com (W.J.D.J.O.); cristinevidal@outlook.com (C.S.V.); 4 Embrapa Mandioca e Fruticultura Tropical, Cruz das Almas 44380-000, Brazil; luciana.oliveira@embrapa.br

**Keywords:** *Manihot esculenta* Crantz, lignin, multivariate statistical analysis, pathway analysis

## Abstract

Cassava is an important staple food for low-income countries. However, its cooking characteristics are especially affected by genotype. In this study, two groups of genotypes, namely hard to cook (HTC) and easy to cook (ETC), were harvested at different times (9 and 15 months), and evaluated by NMR coupled to chemometrics. Additionally, lignin of these materials was studied by ^1^H-^13^C HSQC NMR. The carbohydrates were the most important class of compounds to differentiate the cassava genotypes. The correlation of NMR with cooking time and starch content showed that the higher content of primary metabolites, mostly glucose, can be associated with longer cooking times and reduction of starch, corroborating the metabolic pathways analysis. Furthermore, it was observed that the lignin from cell walls did not differentiate the cooking performance of the genotypes.

## 1. Introduction

Cassava (*Manihot esculenta* Crantz) is well-known for presenting starchy roots that provide calories and nutrition for more than half a billion people [1,2,3]. Cassava is the world’s sixth vital crop because it can be cultivated on marginal soils in diverse conditions including severe drought and high temperatures [1,4,5]. After being boiled or processed, the cassava roots are used as raw material for human and animal nutrition, as well as for energy production [6]. The genetic diversity of cassava represents a broad genomic base for crop breeding programs, which focus on increasing resistance to pests and diseases, adaptation to edaphoclimatic conditions, and the reduction of constraints that limit the yields and quality of cassava [4].

Cassava roots can be harvested anytime from 8 to 24 months after planting [7], and they are an important source of starch, which accounts for 20 to 40% of its weight [8]. In Africa, long-term harvested and bitter cassava is often used for processing, whereas short-term harvested and sweet types are direct boil-and-eat [9]. During the cooking process, starch undergoes modifications as gelatinization, water absorption, and volume increase of the granulates occurs, resulting in a product with relevant characteristics for consumer acceptability [10]. Therefore, the development of cassava cultivars with the most appropriate characteristics for consumers, such as shorter cooking time, is relevant for worldwide population.

Nowadays, emerging metabolomics using advanced analytical techniques such as Nuclear Magnetic Resonance (NMR) spectroscopy allows obtaining comprehensive profiles of the primary metabolism during plant physiological activities [11]. Nevertheless, NMR is not restricted to tissue extract analysis. This technique is suitable for biofluid, intact tissues, organs, and study and characterization of complex materials such as lignin and polysaccharides, among others [12]. Its success derives from advantages such as its inherently nondestructive nature, easy sample preparation, high experimental reproducibility, enabling of the quantification of several metabolites in single ^1^H spectrum, no need for previously chromatographic separation, and allowing for reliable molecule characterization even for unknown compounds [13].

NMR also enables partial characterization of several matrices, such as lignin, which is a hydrophobic material and highly branched natural biopolymer of phenolic monomers [14] and roots [15] in general. Therefore, the aim of this work was to evaluate hard-to-cook (HTC) and easy-to-cook (ETC) cassava genotypes by metabolomics-based fingerprinting through NMR coupled to chemometrics in order to discriminate metabolites associated with the cooking time, and also to associate them with the metabolic pathways to identify metabolic mechanisms associated with cassava hardening. In addition, the lignin portion of the cassava root was analyzed by ^1^H-^13^C HSQC (heteronuclear single quantum correlation) NMR to evaluate the influence of the cell wall material on the cooking time.

## 2. Materials and Methods

### 2.1. Sampling

The cassava genotypes designated as HTC (2009.0213, 2009.0216, 2009.0905 and 2009.1220) and ETC (BRS Brasil, BRS Dourada, Eucalipto and Saracura) were grown at Embrapa Mandioca e Fruticultura (Cruz das Almas-BA, Brazil). The experiment was carried out with plants arranged in a completely randomized design with three replications (8 × 3). A spacing of 1.0 m × 0.7 m fertilized with P_2_O_5_ was used, and the experiment was conducted from May 2016 (planting) to February 2017 (for nine-month harvest) and August 2017 (for fifteen-month harvest). After harvest, the roots from 25 cassava plants were mixed, and a subsample from 20 representative roots was randomly collected for chemical analysis and starch extraction. For cooking evaluation, a total of 12 roots were selected.

For sampling, the roots were washed, chopped into cylinders, peeled, dried, and divided in half lengthwise. Samples from the opposite root sides were used for starch extraction, and the remaining part, which was ground, and 500 g was frozen in an ultrafreezer (at −80 °C), was used for the other analyses.

### 2.2. Lignin Extraction

The lignin extraction was performed by pressurized liquid extraction (Dionex ASE 350, Thermo Fisher Scientific, Waltham, MA, USA). The biological replicates were unified to give rise to one representative genotype sample. Therefore, the lignin was extracted twice for ETC genotypes (BRS Brasil, Saracura, BRS Dourada, Eucalipto) and HTC genotypes (2009.0213, 2009.0216, 2009.0905 and 2009.1220). Dried cassava (5.7 g) was mixed with 1.7 g diatomaceous earth and placed in stainless steel extraction cells of 34 mL. The extractions were performed with 60% ethanol/water (*v*/*v*), acidified with 30 mM H_2_SO_4_, at 190 °C for 75 min. All the extractions were performed in triplicate. The resultant dissolved lignin extract (organosolv liquor) was evaporated and precipitated upon dilution with water (10:1 *w*/*w* dilution ratio H_2_O: organosolv liquor, at 27 °C for 48 h). The lignin was recovered by vacuum filtration using qualitative filter paper of 80 G. The paper with the lignin were dried at 60 °C and weighed to obtain the lignin yield. Then, the resultant samples were evaluated by NMR as described in Section 2.4.

### 2.3. NMR Spectroscopy

We obtained experimental triplicates of each biological replicate (total of three) of eight different cassava genotypes under two different harvest times (nine and 15 months). However, some experimental replicates were not used due to poor spectra quality, resulting in 135 spectra. Therefore, an amount of 30 mg was mixed with 600 μL of a stock solution of D_2_O containing 1 mM of TMSP-d_4_ (sodium-3-trimethylsilylpropionate-2,2,3,3-d_4_). After 2 min at room temperature (24 °C), the solution was centrifuged at 4032× *g* (100 mm rotor, Edulab model 80-2B Centrifuge, Curitiba, Brazil) for 2 min, and the supernatant was transferred to 5 mm NMR tubes. The experiments were performed on an Agilent DD2 equipment operation at 599.56 MHz for ^1^H (14.75 T) and 150.77 MHz for ^13^C, equipped with a 5 mm (^1^H-^19^F/^15^N-^31^P) inverse detection One Probe™ with actively shielded z-gradient.

The ^1^H NMR spectra were acquired in triplicate under quantitative parameters [16,17]: controlled temperature to 299.1 K; hard pulse calibrated to 90° (7.75 μs pulse length at 58 dB of power); acquisition time of 5.0 s and recycling delay of 25.0 s determined by the inversion-recovery pulse sequence. The PRESAT pulse sequence was applied for non-deuterated water suppression, and the spectra were obtained with 24 transients using 48,000 time domain points for a spectral window of 16.0 ppm. The ^1^H NMR spectra processing was performed by applying exponential Lorentzian broadening of 0.3 Hz, and zero filling to 16k points before the Fourier transformation. The phase correction was performed manually, and the automatic baseline correction was applied over the entire spectral range. The spectra were referenced to chemical shift at *δ* 0.0 from TMSP-d_4_ singlet signal.

For compound identification, two-dimensional NMR experiments were acquired using the standard spectrometer library pulse sequences. The ^1^H-^1^H gradient correlation spectroscopy (COSY) experiments were obtained with spectral width of 9615.4 Hz in both dimensions, 1442 × 200 data matrix, 16 scans per t1 increment, and relaxation delay of 1.0 s. The ^1^H-^13^C HSQC experiments were acquired with an evolution delay of 3.425 ms (transfer delay) for a coupling constant one-bond proton-carbon [^1^*J* (C,H)] of 146 Hz, 1442 × 200 data matrix, 48 scans per t1 increment, spectral widths of 9615.4 Hz in f2 and 30,154.5 Hz in f1, and relaxation delay of 1.0 s. The ^1^H-^13^C HMBC (gradient heteronuclear multiple bond correlation) experiments were recorded with an evolution delay of 62.5 ms for a coupling constant ^LR^*J* (C,H) of 8 Hz, 1442 × 200 data matrix, 96 scans per t1 increment, spectral widths of 9615.4 Hz in f2 and 36,182.7 Hz in f1, and relaxation delay of 1.0 s.

### 2.4. Relative Quantitative ^1^H-^13^C HSQC NMR Analysis of the Lignin

An amount of 50 mg of the lignin samples was directly mixed with 600 μL of DMSO-d_6_, inserted into 5 mm NMR tubes, and dipped into an ultrasonic bath for 24 h. The ^1^H-^13^C HSQC NMR analyses were developed with spectral widths of 30,165.9 Hz and 9615.4 for ^13^C and ^1^H dimensions, respectively. They were recorded as 962 complex points with a recycle delay of 0.5 s for ^1^H dimension using 64 transients with 256 complex points for ^13^C dimension, and one-bond ^1^*J* X-H coupling constant of 146 Hz.

The data were processed by means of VNMRJ™ software (version 4.2, Agilent Technologies, Palo Alto, CA, USA). The characteristic signals of the lignin components were assigned in accordance with previously reported data [18,19,20]. A relative quantitative method along with the integrated areas of the HSQC NMR cross peaks from syringyl and guaiacyl were used to determine their proportion.

### 2.5. Chemometric Analysis of the ^1^H NMR Dataset

The ^1^H NMR data were converted to American Standard Code for Information Interchange (ASCII) files and then imported into Origin™ 9.4 software for numerical matrix construction. The spectral region between δ 0.70 and 9.2 was used for chemometric analysis and the area of non-deuterated water suppression (δ 4.60 to 4.94—according to the saturation profile evaluation) was also excluded. The resultant matrix (raw data) was imported into the PLS-Toolbox™ package (version 8.6.2—Eigenvector Research Inc., Manson, WA, USA) under the Matlab™ programming language (R2019a; The MathWorks Inc., Natick, MA, USA) to perform the unsupervised chemometric method by Principal Component Analysis (PCA), as well as supervised methods by Partial Least Squares (PLS) and Partial Least Squares Discriminant Analysis (PLS-DA), with a confidence level of 95%.

Initially, a general evaluation was developed using the total number of spectra, which resulted in a numerical matrix containing 138 cassava samples × 8171 variables in each spectrum, totaling 1,127,598 data points (raw data). In order to detail the cassava discrimination according to the species, additional chemometric analyses were developed considering each harvest time separately, which resulted in two numerical matrices with dimensionality of 563,799 data points: 69 cassava samples × 8171 variables.

Algorithms for baseline correction and normalization were applied over the variables, and variable alignment was performed using COW (Correlation Optimized Warping) with segments of 50 data points and a slack of 5 data points [21]. The sample data were mean-centered, and the Singular Value Decomposition (SVD) algorithm was applied to decompose the original matrices for PCA. For PLS and PLS-DA, the Simplified PLS (SIMPLS) algorithm was applied for modeling, and the number of Latent Variables (LV) was chosen based on the following statistical parameters: RMSEC (Root Mean Squared Error of Calibration); RMSECV (Root Mean Squared Error of Cross Validation); and similarity index (RMSEC/RMSECV) higher than 0.75 [17,22].

### 2.6. Metabolomic Pathway Analysis

In order to evaluate the metabolic pathways associated with different harvesting times (nine and 15 months) and the cooking characteristics (hard and easy to cook) of the aforementioned cassava species, pair-wise comparison was performed for both characteristics by classification model using orthogonal partial least squares discriminant analysis (OPLS-DA using Simplified PLS (SIMPLS) algorithm, venetian blinds w/10 splits and blind thickness = 1), also with the PLS-Toolbox™ software (version 8.6.1., Eigenvector Research Inc., Wenatchee, WA, USA). The OPLS-DA highlights the chemical changes by removing the irrelevant systematic variances using sample classes as reference [23]. Loadings and coefficient plots were analyzed, and the variables important for projection (VIP) with values higher than 1 were quantified and used as input data for metabolic pathway analysis using MetaboAnalyst™ 4.0 (http://www.metaboanalyst.ca, accesses on 30 August 2020) [24,25,26,27].

### 2.7. Determination of Starch (Fresh and Dry Weight) and Cooking Time

Starch analysis was performed in the dry samples according to a well-known methodology [28]. Starches were hydrolyzed by the action of the enzymes α-amylase and amyloglucosidase; and the glucose content quantified by spectrophotometry.

The cooking time was determined using the modified Mattson apparatus. The apparatus consisted of a support formed by two parallel plates with 12 holes, with each supporting 12 cylindrical aluminum connecting rods of 90 g contained in the needle tip. Ten plants per plot were harvested and 10 roots were selected, washed, cut into pieces of 6 cm (cylinders) and peeled. The cylinders were washed and drained, and 12 cylinders were weighed and thermally treated in boiling distilled water (1 kg L^−1^). The cooking time was recorded after 2 cm penetration of needle tips in the 10 cylinders. Softening time was evaluated in two replicates.

## 3. Results and Discussion

### 3.1. Metabolomic Fingerprinting

**Figure 1:**
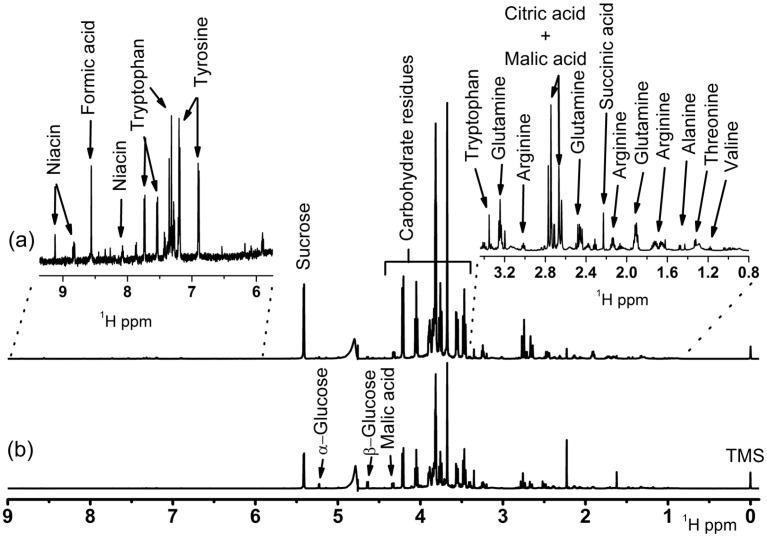
PC1 × PC2 Comparison between the 1H NMR spectra from representative genotypes of cassava harvested after 9 months of planting: (**a**) ETC BRS Dourada and (**b**) HTC hybrid 2009_1220.

Initially, the identification of the main organic compounds in different genotypes of cassava was developed by NMR spectroscopy. Figure 1a,b presents ^1^H NMR spectra from representative genotypes from the ETC cassava harvested after nine months (Dourada) and HTC cassava (2009.1220), respectively. In addition, Table S1 describes structures, ^1^H and ^13^C chemical shifts (*δ*), multiplicity, correlations, coupling constants (*J* in Hz), and references of the correspondent compounds [29,30,31,32,33,34,35,36].

**Figure 2:**
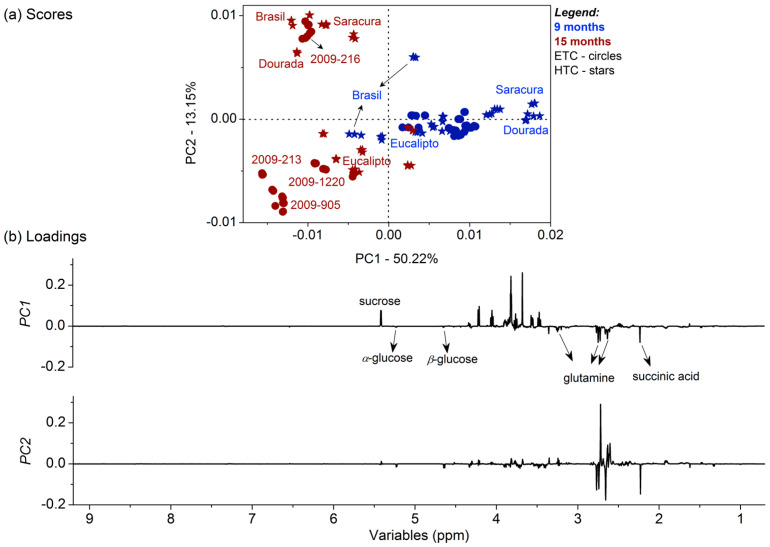
PC1 × PC2 scores coordinate system (**a**) and respective loadings (**b**) of different genotypes of cassava. Legend: cassava genotypes harvested nine months after planting in blue and 15 months after planting in red; HTC genotypes are illustrated as stars and ETC as circles.

It was clear that the compounds detected comprised sugars, amino acids, and short chain organic acids, regardless of the cassava genotypes. In general, slight variations among the compound intensities (concentration) with different cooking characteristics were detected, depending on the genotype. Therefore, due to the complexity of the ^1^H NMR dataset by the elevated number of identified compounds within the cassava genotypes, as well as the inherent similarity among the sample compositions, an unsupervised multivariate statistical evaluation by PCA was applied to investigate the composition variability according to the cassava genotypes related to the harvesting time. Figure 2 presents the PCA results, with the cassava genotypes harvested nine months after planting represented in blue, and 15 months after in red, with HTC genotypes illustrated by stars and ETC genotypes by circles.

The PC1 was the main axis for the cassava separation according to the harvest time, with cassava harvested nine months after planting having positive scores, and those harvested after 15 months having negative scores. It is also observed that the genotypes harvested after 15 months presented higher chemical variation, since samples were more dispersed according to the PC2 axis. The PC1 loadings showed the roots harvested after 15 months had higher contents of glucose, citrate, and succinate, while roots harvested after nine months presented mainly higher contents of sucrose.

**Figure 3:**
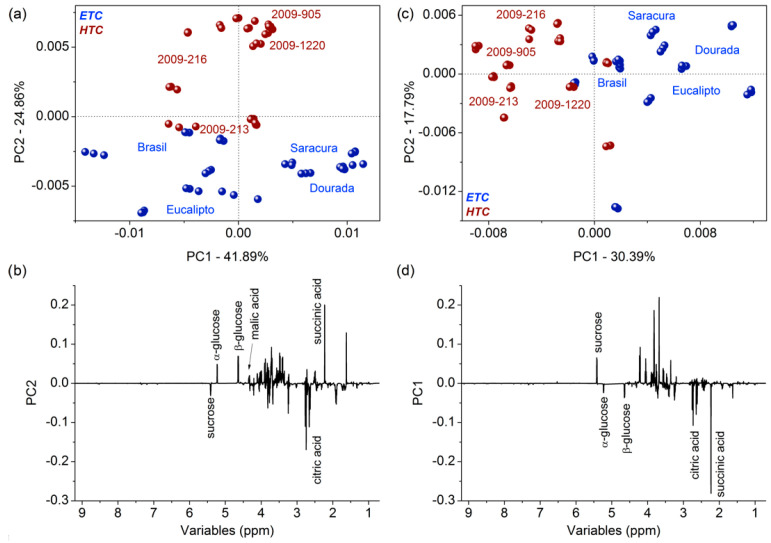
Scores coordinate system (PC1 × PC2) from cassava genotypes harvested nine months after planting (**a**) in blue, and 15 months after planting (**c**) in red. Relevant loadings from cassava genotypes harvested after nine months (**b**) and 15 months (**d**) plotted in lines in the same intensity. HTC genotypes are illustrated as stars, and ETC as circles.

In order to detail the chemical variability of the HTC and ETC genotypes, additional PCA evaluations were performed separately for each genotype, also considering the harvest period (nine or 15 months). Figure 3a,b presents the scores and loadings for genotypes harvested after nine months, respectively; and Figure 3c,d presents the scores and loadings for genotypes harvested after 15 months, respectively. The HTC genotypes are illustrated by stars, and the ETC genotypes by circles.

For genotypes harvested after nine months (Figure 3a), PC2 was the main axis for cassava genotype chemical distinctions according to the cooking performance; for genotypes harvested after 15 months (Figure 3c), the PC1 was the relevant axis. In general, the loadings plots for both harvesting periods (Figure 3b,d) show that ETC cassava genotypes presented higher amounts of sucrose and citrate than the HTC genotypes. On the other hand, the HTC genotypes presented higher amounts of glucose, maleate, and succinate. In addition, for HTC roots harvested after nine months (Figure 3a), a higher content of glucose, maleate, and succinate was observed, and higher content of sucrose and citrate in ETC genotypes with negative scores. For roots harvested after 15 months (blue), the discrepancy in organic compound content was reduced in HTC genotypes that presented higher amounts of glucose, citrate, and succinate, as well as higher amounts of sucrose in ETC genotypes with positive scores (amounts of citrate were inverted from 9 months to 15 months based on HTC and ETC genotypes).

For a comprehensive analysis of cassava harvested with different cooking characteristics, the variables highlighted by the OPLS-DA method were used as input data for metabolic pathway analysis using MetaboAnalyst™ [24,25,26,27,37]. The orthogonalization improved the identification of the chemical variability by removing the unrelated systematic variance according to the sample classes [23]. Table 1 describes the respective statistical parameters of the regressions, and the graphs are available at Support Information File (Figure S1).

**Figure 4:**
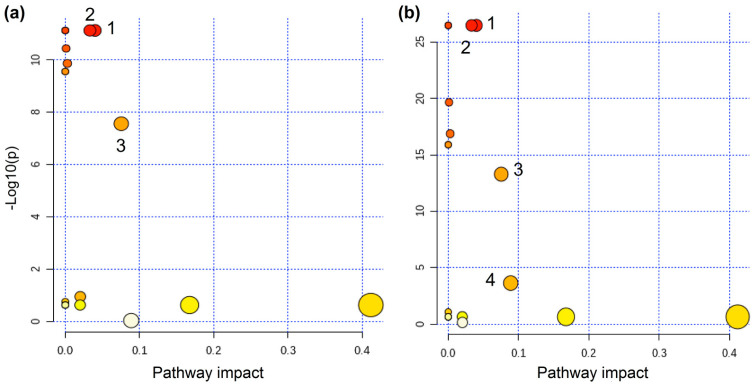
Pathways associated with the metabolism response for HTC and ETC cassava over (**a**) nine months and (**b**) 15 months of harvesting time. Legend: 1—Citrate cycle (TCA cycle); 2—Sulfur metabolism; 3—Galactose metabolism; 4—Starch and sucrose metabolism.

The selected variables, respective chemical shift, and VIP score are shown in Table S2 of the Support Information file. Figure 4a shows the pathways associated with the cooking characteristics for cassava harvested at nine months, and Figure 4b shows the pathway at 15 months. The metabolomic pathways colored from deep red to yellow indicate an increased concentration of metabolites, and the size indicates the impact on pathway based on—log(p). The most significant metabolic pathways with a false discovery rate (FDR) lower than 6.9 × 10^−8^ and 2.33 × 10^−4^ for 9 and 15 months, respectively, and metabolites with impact on the route higher then 0.03 were considered for both harvesting periods: 1 for citrate cycle; 2 for sulfur metabolism; 3 for galactose metabolism; and 4 for starch and sucrose metabolism (for more detail of the pathways selection, see Table S3 at the Support Information file).

Different pathways associated with cooking characteristics of the cassava roots were similar regardless of harvesting time (Figure 3). The lower content of succinate in ETC roots might induce the downregulation of the citrate cycle (TCA cycle or Krebs cycle) (1) and sulfur metabolism (2). The TCA cycle provides the carbon skeleton for biosynthesis of several compounds [38]; sulfur metabolism is essential for plant growth, development, and response to environmental changes. Therefore, the suppression of both pathways might affect aerial plant growth [39]. The lower content of glucose in the ETC roots also might induce the downregulation of galactose metabolism (3), which is linked to the synthesis of raffinose family oligosaccharides (RFOs) [40]. The RFOs protect plant cells from oxidative damage caused by various types of stress conditions [41,42], and act as carbon transport and storage [43], which also might affect plant development. Finally, upregulation of sucrose in the ETC roots was observed. Sucrose metabolism is linked to starch and sucrose metabolism (4) changing properties of starch in grains, and the upregulation might induce accumulation of starch in the root [44].

Free sugars were the most important components to differentiate the genotypes and their cooking performance. They were directly correlated with starch and sucrose metabolism (Figure 4), and the roots with higher content of glucose tended to present reduced content of starch and higher cooking time. Therefore, in order to corroborate this information, the content of starch (at fresh and dried base) and the cooking performance of the roots were obtained; the results are presented in Table S4. Seeking to correlate the important information obtained by NMR with those variables, a multivariate regression modeling by PLS was developed using the cooking time and percentage of starch (fresh and dry weight) as independent variables. This information is important because a good model indicates that we can perform a direct correlation of the root hardening with cooking performance, starch content, and free sugar content. 

The regression modeling was developed for genotypes from the different harvesting periods (9 and 15 months) separately, maximizing the covariance between the dependent variables (^1^H NMR dataset) and the independent variables (cooking time and percentage of starch). Table 2 describes the respective statistical parameters of the regressions.

In general, the models presented high correlation coefficients of calibration (R^2^ above 0.9) and validation (R^2^ above 0.8), very low bias values (non-biased models), and relatively low calibration and validation errors achieved by the RMSEC and RMSECV methods with elevated similarity criterion (proximity between RMSEC and RMSECV values) [13,18]. Despite the satisfactory results represented by the figures of merit, the cooking time model presented higher calibration and cross-validation errors with lower correlation coefficients. This fact is related to the cooking procedures that were interrupted at 50 min (standard time for cooking essay) and, consequently, the genotypes with longer cooking times might have this parameter underestimated.

In general, the models presented were well adjusted according to the independent variables related to the cooking time for cassava genotypes at different harvesting times (9 and 15 months). The compositional variability of the entire ^1^H NMR spectra showed a close correlation among the composition and cooking characteristics of the root. Therefore, the study of the correlation of NMR and the starch accumulation at the roots is essential, since starch and sucrose metabolism pathways were triggered. The models show that there is a correlation between NMR spectrum and starch content of the root: for both variables (percentage of starch at fresh and dry weight), the model for nine months was better adjusted than for 15 months. It was previously revealed by the PCA evaluations (Figure 2 and Figure 3) that cassava harvested after 15 months presents higher contents of glucose, while roots harvested at nine months mainly present higher contents of sucrose. The free sugar accumulation in cassava was correlated with disruption in the starch synthesis pathway by enzyme activity [45]. Glucose was found to be the major free sugar in cassava, with low starch content and reduced levels of amylose [45].

In starch biosynthesis, the enzyme granule-bound starch synthase I (GBSSI) polymerizes amylose from the donor substrate ADP-glucose [46]. The inhibition of this enzyme produces amylose-free starches, which melt at a higher temperature and lead to weaker gels [47]. Therefore, the lower content of glucose along with higher content of sucrose in ETC roots might be correlated to higher contents of starch in these roots that possess better cooking characteristics. In addition, these data shows that the roots harvested at nine months might possess higher contents of starch and better cooking characteristics.

### 3.2. Relative Quantitative ^1^H-^13^ HSQC NMR Analysis of the Lignin in Cassava Genotypes

**Figure 5:**
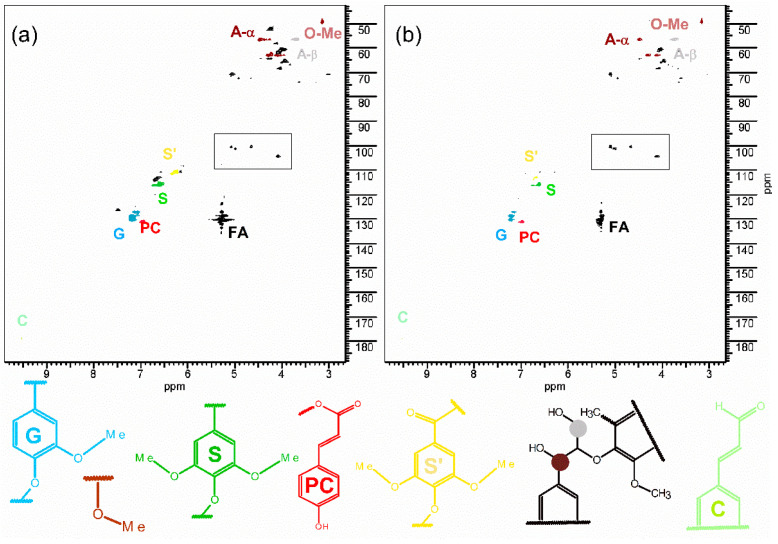
^1^H-^13^C HSQC of the lignin from cassava extracted in (**a**) genotype BRS Dourada (ETC); (**b**) hybrid 2009 12–20 (HTC). Legend: S: syringyl; S’: oxidized syringyl; G: guaiacyl; O-Me: methoxyl groups; PC: p-coumarate; C: cinnamic aldehyde; A: aryl ether with A-α: α–*O*–4 and A-β: β–*O*–4; FA: fatty acids bonded to lignin; square region: polysaccharides bonded to lignin.

In order to evaluate the influence of the cell wall material on cooking time, lignin of the genotypes with different cooking characteristics was analyzed. Figure 5a shows the representative ^1^H-^13^ HSQC NMR spectrum of the lignin extracted from BRS Dourada (ETC), and Figure 5b shows the hybrid 2009.1220 (HTC) lignin.

The presence of the main compounds from lignin was detected as syringyl (characteristic signals ^1^H/^13^C: 6.60/115.4 ppm) and guaiacyl (characteristic signals ^1^H/^13^C: 7.14/126.9; 7.20/128.5; 7.20/129.7 ppm). In addition, the oxidized syringyl (characteristic signals ^1^H/^13^C: 6.34/111.2 ppm), p-coumarate (characteristic signals ^1^H/^13^C: 6.96/130.8 ppm), cinnamic aldehyde (characteristic signals ^1^H/^13^C: 9.52/178.5 ppm), and polysaccharides residues as aryl ether linkage as α–*O*–4 (A-α characteristic signals ^1^H/^13^C: 4.42/56.7 ppm), β–*O*–4 (A-β characteristic signals ^1^H/^13^C: 4.01/60.5 ppm), and the anomeric signal were observed, highlighted by square in Figure 5a,b. The ratio of the syringyl and guaiacyl was also evaluated for the genotypes with different cooking characteristics. The information covering the syringyl and guaiacyl ratio is a relevant parameter for matrix understanding: lignin with a higher amount of syringyl is more easily removed during the delignification process [48], since the lesser reactivity of the C5 aromatic carbon from syringyl implies a less-condensed structure which increases the lignin solubility [49]. Therefore, the high content of guaiacyl might impose a greater constraint in the matrix, inducing a stiffening of the system. Table 3 displays the syringyl and guaiacyl ratio of the lignin obtained from the different genotypes, where the syringyl and guaiacyl ratio did not change with cooking characteristics or genotype of cassava. Therefore, the cooking performance of the root might be more associated to the disruption in the starch synthesis pathway than the cell wall adhesive characteristics (lignin).

## 4. Conclusions

The choice of harvesting time and genotypes plays an important role on cooking performance and, consequently, in the final destination of the cassava roots. Roots harvested after 15 months present higher contents of glucose, citrate, and succinate, while roots harvested nine months after planting mainly present higher contents of sucrose. In general, regardless of the harvesting time, the easy-to-cook cassava genotypes presented higher amounts of sucrose and lower amounts of glucose than hard-to-cook genotypes. Metabolomic variability among the cassava genotypes according to the harvest period and cooking time was associated with important pathways, such as galactose, sucrose, and starch metabolisms. Roots with higher contents of glucose present reduced contents of starch and higher cooking times. Since starch is a gelatinization agent, its low content can be associated with longer cooking times, which was corroborated with the trigged pathways and the multivariate regression of the ^1^H NMR data, as well as the percentage of starch. Therefore, clones harvested after nine months and those designated as easy to cook (Saracura, Dourada, Eucalipto, and Brasil) might possess better cooking characteristics. It was also observed that the syringyl/guaiacyl ratio in lignin did not correlate to the cooking performance of the genotypes. Therefore, this study contributes to the understanding of the biosynthetic mechanism leading to the different cooking characteristics of cassava.

## Figures and Tables

**Table 1:** Statistical parameters of the multivariate regression from OPLS-DA modeling of the cooking characteristics at 9 and 15 months.

Cooking Characteristics							
Model	2 LV ^a^ (%)	Bias ^b^	r^2^ cal ^c^	RMSEC ^d^	r^2^ val ^e^	RMSECV ^f^	RMSEC/RMSEV ^g^
9 months	61.82	−1.03 × 10^−3^	0.80	0.22	0.80	0.22	1
15 months	92.54	−4.29 × 10^−3^	0.92	0.14	0.91	0.15	0.93

^a^ The total variance percent in the X matrix refers to the first two Latent Variable (LV); ^b^ Influenced modeling; ^c^ Coefficient of correlation of the calibration; ^d^ Root Mean Square Error of Calibration; ^e^ Coefficient of correlation of the validation; ^f^ Root Mean Square Error of the Cross Validation; ^g^ Similarity criterion.

**Table 2:** Statistical parameters of the multivariate regression from PLS modeling of the cooking time, percentage of starch at fresh root, and starch at dried base according to the cassava aging.

Cooking Time							
Model	5 LV ^a^ (%)	Bias ^b^	r^2^ cal ^c^	RMSEC ^d^	r^2^ val ^e^	RMSECV ^f^	RMSEC/RMSEV ^g^
9 months	88.35	−1.4 × 10^−14^	0.91	3.31	0.86	4.00	0.83
15 months	74.17	−7.1 × 10^−15^	0.91	3.42	0.88	4.10	0.83
Percentage of starch at fresh root							
Model	8 LV ^a^ (%)	Bias ^b^	r^2^ cal ^c^	RMSEC ^d^	r^2^ val ^e^	RMSECV ^f^	RMSEC/RMSEV ^g^
9 months	86.74	−3.5 × 10^−15^	0.96	0.70	0.88	1.27	0.55
15 months	84.95	0	0.98	0.57	0.96	0.92	0.62
Starch at dried base							
Model	8 LV ^a^ (%)	Bias ^b^	r^2^ cal ^c^	RMSEC ^d^	r^2^ val ^e^	RMSECV ^f^	RMSEC/RMSEV ^g^
9 months	87.33	−2.8 × 10^−14^	0.96	1.17	0.88	2.06	0.57
15 months	82.80	−2.8 × 10^−14^	0.98	1.20	0.94	1.92	0.62

^a^ The total variance percent in the X matrix refers to the first five Latent Variable (LV); ^b^ Influenced modeling; ^c^ Coefficient of correlation between the real times to cook and those predicted during the calibration; ^d^ Root Mean Square Error of Calibration; ^e^ Coefficient of correlation between the real times to cook and those predicted during the validation; ^f^ Root Mean Square Error of the Cross Validation; ^g^ Similarity criterion.

**Table 3:** Ratio of syringyl/guaiacyl (S/G) from the different cassava genotypes.

Genotype	S/G
Saracura	0.6255
BRS Dourada	0.8385
Eucalipto	0.8655
BRS Brasil	0.79321
2009.0213	0.8395
2009.0216	0.8513
2009.0905	0.7965
2009.1220	0.8320

## Data Availability

The data presented in this study are available in this article and supplementary materials.

## Supplementary Materials

The following are available online at https://www.mdpi.com/article/10.3390/foods11111651/s1, Figure S1: LV scores and VIP graphs from OPLS-DA model for HTC and ETC cassava harvested at 9 (a and b) and 15 months (c and d), Table S1: Organic compounds identified in the cassava roots, Table S2. Selected variables with their respective chemical shift selected for quantification and the VIP scores, Table S3. Pathway analysis report for HTC versus ETC for cassava harvested after 9 months and 15 months. The pathway in bold were selected, Table S4. Samples used for NMR analysis and respective raw data for percentage of starch at fresh root, starch at dried base, and cooking time.
