# Supplementary material for: NMR-Based Metabolomic Approach for Evaluation of the Harvesting Time and Cooking Characteristics of Different Cassava Genotypes
Source: Foods. 2022 Jun 3;11(11):1651. doi: 10.3390/foods11111651 (PMC9180251; doi:10.3390/foods11111651)
Supplement: Supplementary file 1 [file foods-11-01651-s001.zip › foods-1741308-supplementary.pdf]

Supplementary Materials

NMR data from the identification of the organic compounds

Tables S1 show the structures. ^{1}H and ^{13}C chemical shifts (*δ*). multiplicity. correlations. and constant coupling (*J* in HZ) of the compounds identified in the cassava roots (Alves Filho, Sartori, Silva, Silva, Fadini, Soong, et al., 2015; Alves Filho, Sartori, Silva, Venâncio, Carneiro, & Ferreira, 2015; Alves Filho, Silva, Teofilo, Larsen, & de Brito, 2017; Balayssac, Trefi, Gilard, Malet-Martino, Martino, & Delsuc, 2009; Davis, Cai, Davies, & Lewis, 1996; Nord, Vaag, & Duus, 2004; Wishart, Jewison, Guo, Wilson, Knox, Liu, et al., 2012; Ye, Yang, Lou, Chen, Yan, & Tang, 2014).

Table S1. Organic compounds identified in the cassava roots.

Structures	*δ* ^{1}H *(multip.* J in Hz)*	*δ* ^{13}C *(HSQC)*	*δ* ^{1}H *ref.*	*δ* ^{13}C *ref.*
	AMINO ACIDS			
Alanine 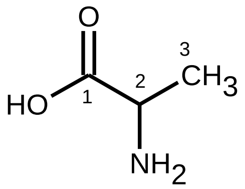	2 - (o)	52.4	3.90 (*q* 7.3)	53.4
	3 - 1.49 (*d* 7.2)	19.9	1.52 (*d* 7.3)	19.1
Glutamine 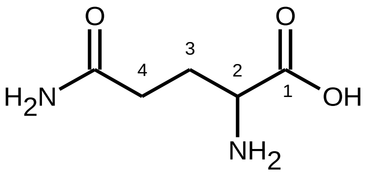	4 - 2.44 to 2.50 (*m*)	34.0	2.45 (*m*)	33.9
	3 - 1.89 a 1.95 (*m*)	30.7	2.12 (*m*)	29.3
	2 - 3.20 (o)	57.1	3.77 (o)	57.2
Threonine 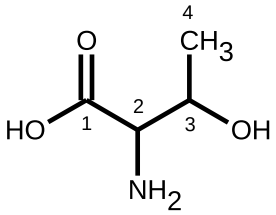	2 – 3.9 (o)	62.2	3.81 (d 4.2)	63.4
	3 – 4.3 (o)	70.0	4.35 (m)	69.3
	4 – 1.34 (d 6.6)	22.8	1.35 (d 6.5)	22.3
Arginine 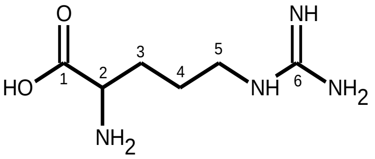	4 – 1.70 (m)	26.7	1.69 (m)	26.5
	3 – 2.10 (m)	29.2	1.90 (m)	30.2
	5 – 3.03	42.5	3.23	43.1
	2 – o	56.0	3.75	56.9
Tyrosine 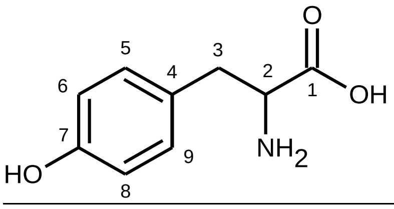	6.8 - 6.91 (*m*)	118.8	6.89 (*m*)	118.9
	5.9 - 7.20 (*m*)	133.7	7.19 (*m*)	133.5
	2 - (o)	no	3.93 (*dd*)	59.0
	3 - 3.20 (o)	39.6	3.06 (*dd*)	38.3
		128.0	7.30 (*s*)	127.9
	2 - 7.33 (*m*)	125.2	7.27	124.9
Tryptophan	4 - 7.29 (*m*)	122.5	7.19	122.2
	5 - 7.21 (*m*)	121.5	7.71	121.2
	6 - 7.74 (*m*)	115.2	7.53	114.7

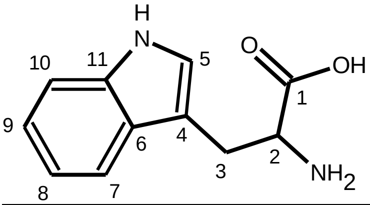	7 - 7.55<br>8 - 3.61<br>9 - 3.36<br>10 – no	29.2<br>29.2<br>no	3.29<br>2.46<br>4.03	29.1<br>29.1<br>57.9
ORGANIC ACIDS				
Malic<br>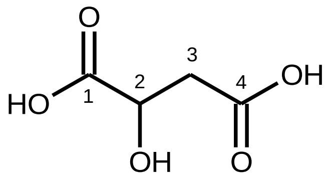	3 - 2.40 (*m*)<br>3 - 2.70 (*m*)<br>2 - 4.31 (*m*)	45.2<br>45.2<br>73.2	2.68 (*dd*)<br>2.85 (*dd*)<br>4.28 (*m*)	45.5<br>45.5<br>73.2
Citric<br>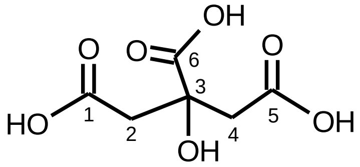	2 - 2.62 (o)<br>4 - 2.74 (o)	47.7<br>47.7	2.52 (*d* 15.8)<br>3.66 (*d* 15.8)	48.6<br>48.6
Succinic<br>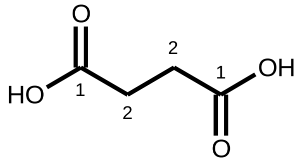	2;3 - 2.41 (s)	37.5		
Acetic<br>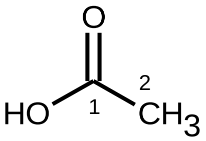	2 - 1.94 (*s*)	27.0	1.90 (*s*)	26.1
Formic<br>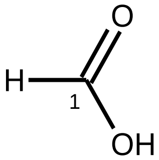	1 - 8.46 (*s*)	no	8.46 (*s*)	173.9
CARBOHYDRATES				
α-glucose<br>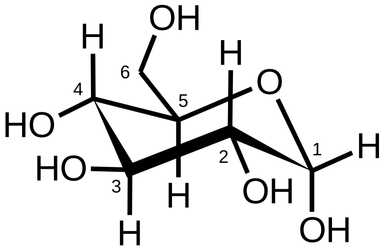	1 - 5.24 (*d* 3.8)<br>2 - 3.46 (m)<br>3 - 3.77 (m)<br>4 - 3.55 (m)<br>5 - 3.71 (m)<br>6 - 3.86 (m)	95.0<br>72.4<br>75.6<br>74.1<br>63.8<br>75.5	5.25 (*d* 3.80)<br>3.89-3.36 (o)<br>n<br>n<br>n<br>n	95.4<br>72.2<br>76.0<br>72.8<br>64.2<br>74.5
β-glucose<br>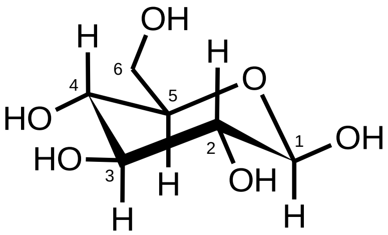	1 - 4.67 (*d* 7.90)<br>2 - 3.26 (m)<br>3 - 3.77 (m)<br>4 - 3.46 (m)<br>5 - 3.43 (m)<br>6 - 3.91 (m)	98.7<br>77.4<br>63.6<br>78.9<br>72.1<br>63.5	4.66 (*d* 8.10)<br>3.25 (*t* 8.40)<br>n<br>n<br>n<br>n	99.2<br>77.6<br>56.1<br>79.0<br>72.8<br>63.1

Sucrose 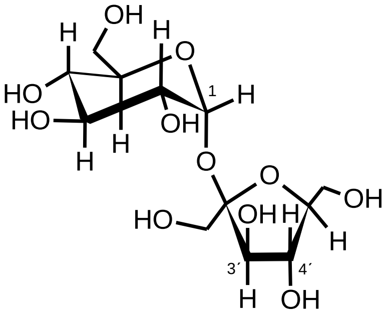	1 - 5.41 (*d* 3.70)	95.1	5.44 (*d* 3.80)	94.7
	2 - 3.56 (o)	74.1	3.89-3.57 (*m*)	73.5
	3 - 3.76 (o)	75.5	n	75.0
	4 - 3.48 (o)	72.3	n	71.8
	5 - 3.85 (o)	75.5	n	74.9
	6 - 3.82 (o)	63.1	n	62.8
	1′ - 3.82 (o)	65.2	n	64.0
	2′ - 3.89 (o)	84.3	n	83.7
	3′ - 4.05 (*m*)	77.0	4.08 (*t* 8.40)	76.6
	4′ - 4.22 (*m*)	79.3	4.24 (*d* 9.0)	79.0
	6′ - 3.68 (*m*)	64.5	n	65.0
	OTHER COMPOUNDS			
Niacin 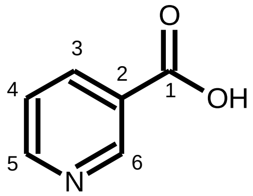	3 – 9.10	no	8.97	152.8
	4 – 8.83	no	8.61	151.4
	5 – 8.07	no	7.54	123.3
	6 – 8.80	no	8.26	145.6

s – simplet; *d* – duplet; *t* – triplet; *q* – quadruplet; *quin* – quintet; *dd* – double of duplets; *dt* – double of triplets; o – overlapping signal; n – no information; no – not observed.

For comprehensive analysis of cassava harvested at 9 and 15 months and with different cooking characteristics. the variables were highlighter by the OPLS-DA and the results are shown for 9 month at Figure 1SIa (LV graph) and Figure 1SIb (resultant VIP score graph) for 15 month at Figure 1SIc (LV graph) and Figure 1SId (resultant VIP score graph).

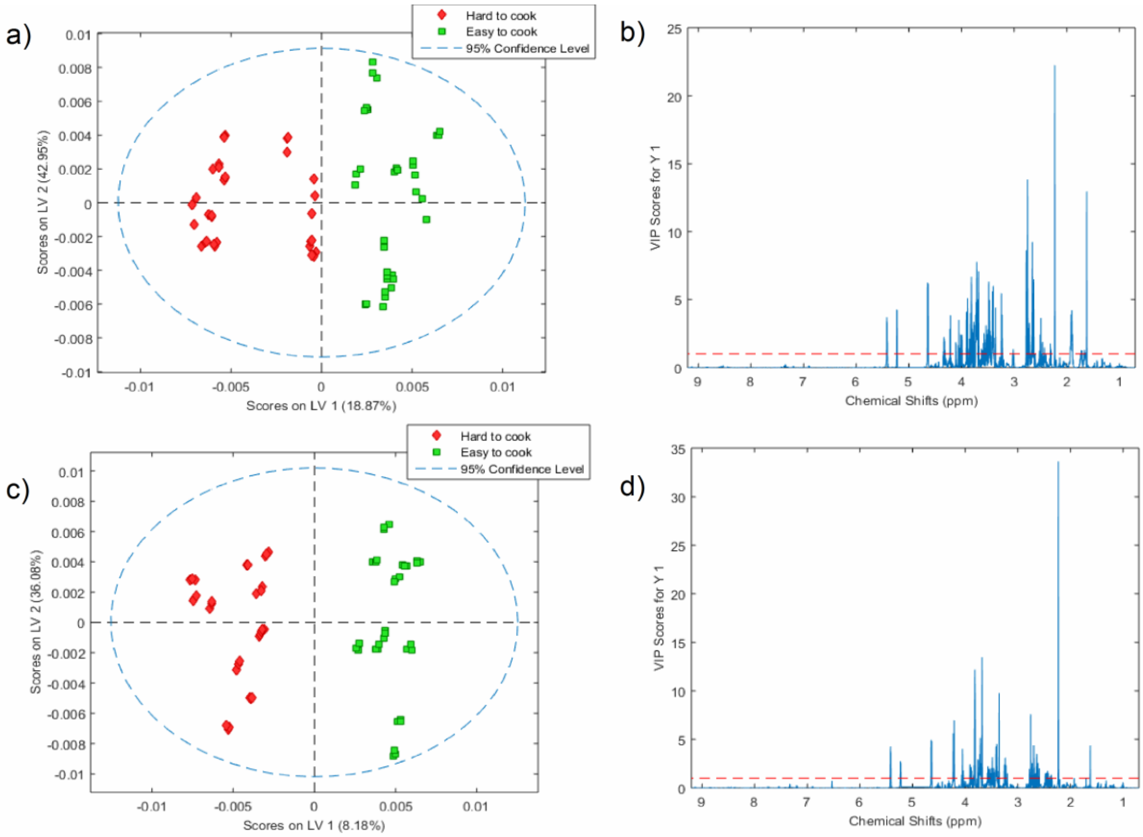

Figure S1. LV scores and VIP graphs from OPLS-DA model for HTC and ETC cassava harvested at 9 (a and b) and 15 months (c and d).

The Table S2 above shows the selected variables with their respective chemical shift selected for quantification and the VIP scores and Table 4SI shows the results for pathway analysis for HTC versus ETC for cassava harvested after 9 months and 15 months.

Table S2. Selected variables with their respective chemical shift selected for quantification and the VIP scores.

Molecule	Chemical Shift (ppm)	VIP score
Succinic acid	2.41	1.5
Glutamine	1.89 a 1.95	4.2
Malic acid	2.70	13.8
β-glucose	4.67	6.2
α-glucose	5.24	4.3
Sucrose	5.41	3.7

The Table S3 presents the report for pathway analysis.

Table S3. Pathway analysis report for HTC versus ETC for cassava harvested after 9 months and 15 months. The pathway in bold were selected.

**HTC versus ETC for 9 months**					
**Pathway Name**	**Match Status**	**p**	**-log(p)**	**FDR**	Impact
**Citrate cycle (TCA cycle)**	**1/20**	**1.89535E-08**	**11.12**	**2.88E-07**	**0.0401**
**Sulfur metabolism**	**1/15**	**1.89535E-08**	**11.12**	**2.88E-07**	**0.0331**
Alanine. aspartate and glutamate metabolism	1/22	1.89535E-08	11.12	2.88E-07	0.0
Propanoate metabolism	1/20	1.89535E-08	11.12	2.88E-07	0.0
Butanoate metabolism	1/17	1.89535E-08	11.12	2.88E-07	0.0
**Glycolysis/Gluconeogenesis**	**2/26**	**9.22275E-08**	**10.433**	**1.17E-06**	**0.0011**
**Glyoxylate and dicarboxylate metabolism**	**2/29**	**3.4435E-07**	**9.861**	**3.74E-06**	**0.0028**
Fructose and mannose metabolism	1/20	7.0045E-07	95.526	5.92E-07	0.0
Amino sugar and nucleotide sugar metabolism	1/50	7.0045E-07	95.526	5.92E-07	0.0
**Galactose metabolism**	**2/27**	**6.8945E-08**	**75.594**	**5.24E-04**	**0.0754**
Nicotinate and nicotinamide metabolism	1/13	0.10954	0.96041	0.18921	0.0202
Nitrogen metabolism	1/12	0.17059	0.76805	0.24237	0.0
Isoquinoline alkaloid biosynthesis	1/6	0.22962	0.63899	0.24237	0.4118
Tyrosine metabolism	1/18	0.22962	0.63899	0.24237	0.1657
Phenylalanine. tyrosine and tryptophan biosynthesis	1/22	0.22962	0.63899	0.24237	0.0200
Ubiquinone and other terpenoid-quinone biosynthesis	1/35	0.22962	0.63899	0.24237	0.0
Phenylpropanoid biosynthesis	1/35	0.22962	0.63899	0.24237	0.0
Aminoacyl-tRNA biosynthesis	1/46	0.22962	0.63899	0.24237	0.0

Starch and sucrose metabolism	1/22	0.89896	0.04626	0.89896	0.0889

HTC versus ETC for 15 months					
Pathway Name	Match Status	p	-log(p)	FDR	Impact
**Citrate cycle (TCA cycle)**	**1/20**	**3.40E-24**	**26.468**	**1.29E-22**	**0.0401**
**Sulfur metabolism**	**1/15**	**3.40E-24**	**26.468**	**1.29E-22**	**0.0331**
Alanine. aspartate and glutamate metabolism	1/22	3.40E-24	26.468	1.29E-22	0.0
Propanoate metabolism	1/20	3.40E-24	26.468	1.29E-22	0.0
Butanoate metabolism	1/17	3.40E-24	26.468	1.29E-22	0.0
**Glycolysis/Gluconeogenesis**	**2/26**	**2.24E-17**	**19.65**	**7.08E-16**	**0.0011**
**Glyoxylate and dicarboxylate metabolism**	**2/29**	**1.35E-13**	**16.871**	**3.66E-14**	**0.0028**
Fructose and mannose metabolism	1/20	1.28E-12	15.893	2.70E-12	0.0
Amino sugar and nucleotide sugar metabolism	1/50	1.28E-12	15.893	2.70E-12	0.0
**Galactose metabolism**	**2/27**	**5.22E-10**	**13.283**	**9.91E-10**	**0.0754**
**Starch and sucrose metabolism**	**1/22**	**2.33E-04**	**36.321**	**4.03E+00**	**0.0889**
Nitrogen metabolism	1/12	0.084765	10.718	0.13421	0.0
Isoquinoline alkaloid biosynthesis	1/6	0.22785	0.64236	0.2405	0.4118
Tyrosine metabolism	1/18	0.22785	0.64236	0.2405	0.1676
Phenylalanine. tyrosine and tryptophan biosynthesis	1/22	0.22785	0.64236	0.2405	0.0200
Ubiquinone and other terpenoid-quinone biosynthesis	1/35	0.22785	0.64236	0.2405	0.0
Phenylpropanoid biosynthesis	1/35	0.22785	0.64236	0.2405	0.0
Aminoacyl-tRNA biosynthesis	1/46	0.22785	0.64236	0.2405	0.0
Nicotinate and nicotinamide metabolism	1/13	0.71764	0.1441	0.71764	0.0202

At Table S4 is presented the samples used for NMR analysis and respective raw data for percentage of starch at fresh root, starch at dried base, and cooking time.

Table S4. Samples used for NMR analysis and respective raw data for percentage of starch at fresh root, starch at dried base, and cooking time.

**Samples**	**Percentage of starch at fresh root**	**Starch at dried base**	**Cooking time**
20090213BI_9m_01	28	89.2	50
20090213BI_9m_02	28	89.2	50
20090213BI_9m_03	28	89.2	50
20090213BII_9m_01	23.31	79.15	50
20090213BII_9m_02	23.31	79.15	50

20090213BII_9m_03	23.31	79.15	50
20090213BIII_9m_01	26.88	85.23	50
20090213BIII_9m_02	26.88	85.23	50
20090213BIII_9m_03	26.88	85.23	50
20090216BI_9m_01	22.63	91.8	50
20090216BI_9m_02	22.63	91.8	50
20090216BI_9m_03	22.63	91.8	50
20090216BII_9m_01	21.21	90.44	50
20090216BII_9m_02	21.21	90.44	50
20090216BII_9m_03	21.21	90.44	50
20090216BIII_9m_01	26.7	93.69	50
20090216BIII_9m_02	26.7	93.69	50
20090216BIII_9m_03	26.7	93.69	50
20090905BII_9m_01	25.49	90.74	50
20090905BII_9m_02	25.49	90.74	50
20090905BII_9m_03	25.49	90.74	50
20090905BIII_9m_01	26	87.44	50
20090905BIII_9m_02	26	87.44	50
20090905BIII_9m_03	26	87.44	50
20091220BI_9m_01	23.57	78.25	50
20091220BI_9m_02	23.57	78.25	50
20091220BI_9m_03	23.57	78.25	50
20091220BII_9m_01	30.33	85.28	50
20091220BII_9m_02	30.33	85.28	50
20091220BII_9m_03	30.33	85.28	50
20091220BIII_9m_01	25.25	65.48	50
20091220BIII_9m_02	25.25	65.48	50
20091220BIII_9m_03	25.25	65.48	50
BrasilBI_9m_01	23.15	84.4	25.1
BrasilBI_9m_02	23.15	84.4	28.1
BrasilBI_9m_03	23.15	84.4	26.6
BrasilBII_9m_01	24.33	84.14	33.1
BrasilBII_9m_02	24.33	84.14	33.1
BrasilBII_9m_03	24.33	84.14	33.1
BrasilBIII_9m_01	22.93	84.75	48.3
BrasilBIII_9m_02	22.93	84.75	50
BrasilBIII_9m_03	22.93	84.75	49.15
DouradaBI_9m_01	24.02	83.46	50
DouradaBI_9m_02	24.02	83.46	46.3
DouradaBI_9m_03	24.02	83.46	48.15
DouradaBII_9m_01	26.18	82.53	50
DouradaBII_9m_02	26.18	82.53	50
DouradaBII_9m_03	26.18	82.53	50
DouradaBIII_9m_01	24.31	83.62	50
DouradaBIII_9m_02	24.31	83.62	50
DouradaBIII_9m_03	24.31	83.62	50
EucaliptoBI_9m_01	32.88	90.09	16.5
EucaliptoBI_9m_02	32.88	90.09	16.2
EucaliptoBI_9m_03	32.88	90.09	16.35
EucaliptoBII_9m_01	33.17	91.94	28.4
EucaliptoBII_9m_02	33.17	91.94	23.2
EucaliptoBII_9m_03	33.17	91.94	25.8
EucaliptoBIII_9m_01	33.45	89.88	20.4

EucaliptoBIII_9m_02	33.45	89.88	19.5
EucaliptoBIII_9m_03	33.45	89.88	19.95
SaracuraBI_9m_01	30.94	91.22	48.1
SaracuraBI_9m_02	30.94	91.22	26.8
SaracuraBI_9m_03	30.94	91.22	37.45
SaracuraBII_9m_01	30.72	91.87	50
SaracuraBII_9m_02	30.72	91.87	44.1
SaracuraBII_9m_03	30.72	91.87	47.05
SaracuraBIII_9m_01	31.97	95.73	50
SaracuraBIII_9m_02	31.97	95.73	50
SaracuraBIII_9m_03	31.97	95.73	50
20090213BI_15m_01	30.58	85.81	38.4
20090213BI_15m_02	30.58	85.81	38.4
20090213BI_15m_03	30.58	85.81	38.4
20090213BII_15m_01	30.38	83.6	28.5
20090213BII_15m_02	30.38	83.6	28.5
20090213BII_15m_03	30.38	83.6	28.5
20090216BI_15m_01	24.1	70.37	32.1
20090216BI_15m_02	24.1	70.37	32.1
20090216BI_15m_03	24.1	70.37	32.1
20090216BII_15m_01	28.5	77.04	38.4
20090216BII_15m_02	28.5	77.04	38.4
20090216BII_15m_03	28.5	77.04	38.4
20090216BIII_15m_01	26.11	73.29	48.5
20090216BIII_15m_02	26.11	73.29	48.5
20090216BIII_15m_03	26.11	73.29	48.5
20090905BI_15m_01	23.77	63.44	36.1
20090905BI_15m_02	23.77	63.44	36.1
20090905BI_15m_03	23.77	63.44	36.1
20090905BII_15m_01	29.61	81.9	50
20090905BII_15m_02	29.61	81.9	50
20090905BII_15m_03	29.61	81.9	50
20090905BIII_15m_01	25.71	72.07	50
20090905BIII_15m_02	25.71	72.07	50
20090905BIII_15m_03	25.71	72.07	50
20091220BI_15m_01	33.15	81.1	42.6
20091220BI_15m_02	33.15	81.1	42.6
20091220BI_15m_03	33.15	81.1	42.6
20091220BII_15m_01	30.77	72.94	50
20091220BII_15m_02	30.77	72.94	50
20091220BII_15m_03	30.77	72.94	50
20091220BIII_15m_01	30.3	70.75	50
20091220BIII_15m_02	30.3	70.75	50
20091220BIII_15m_03	30.3	70.75	50
BrasilBI_15m_01	28.97	88.27	19.4
BrasilBI_15m_02	28.97	88.27	18.2
BrasilBI_15m_03	28.97	88.27	18.8
BrasilBII_15m_01	34.3	90.75	17.3
BrasilBII_15m_02	34.3	90.75	14.3
BrasilBII_15m_03	34.3	90.75	15.8
BrasilBIII_15m_01	36.18	90.46	17.1
BrasilBIII_15m_02	36.18	90.46	21.3
BrasilBIII_15m_03	36.18	90.46	19.2

DouradaBI_15m_01	27.72	84.75	23.5
DouradaBI_15m_02	27.72	84.75	18.2
DouradaBI_15m_03	27.72	84.75	20.85
DouradaBII_15m_01	28.99	83.71	27.4
DouradaBII_15m_02	28.99	83.71	32.2
DouradaBII_15m_03	28.99	83.71	29.8
DouradaBIII_15m_01	26.19	84.51	35.4
DouradaBIII_15m_02	26.19	84.51	24.3
DouradaBIII_15m_03	26.19	84.51	29.85
EucaliptoBII_15m_01	32.82	79.1	20.1
EucaliptoBII_15m_02	32.82	79.1	16.5
EucaliptoBII_15m_03	32.82	79.1	18.3
EucaliptoBIII_15m_01	37.64	85.05	29
EucaliptoBIII_15m_02	37.64	85.05	29.2
EucaliptoBIII_15m_03	37.64	85.05	29.1
SaracuraBI_15m_01	38.28	92.34	14.3
SaracuraBI_15m_02	38.28	92.34	27.3
SaracuraBI_15m_03	38.28	92.34	20.8
SaracuraBII_15m_01	39.44	92.32	24.3
SaracuraBII_15m_02	39.44	92.32	22.1
SaracuraBII_15m_03	39.44	92.32	23.2
SaracuraBIII_15m_01	39.2	91.34	26.5
SaracuraBIII_15m_02	39.2	91.34	28.6
SaracuraBIII_15m_03	39.2	91.34	27.55

References

1. Alves Filho, E. G., Sartori, L., Silva, L., Silva, B. F., Fadini, P. S., Soong, R., Simpson, A., & Ferreira, A. G. (2015). Non-targeted analyses of organic compounds in urban wastewater. *Magnetic Resonance in Chemistry, 53*(9), 704-710. https://doi.org/10.1002/mrc.4169.
2. Alves Filho, E. G., Sartori, L., Silva, L., Venâncio, T., Carneiro, R. L., & Ferreira, A. G. (2015). 1H qNMR and Chemometric Analyses of Urban Wastewater. *Journal of the Brazilian Chemical Society, 26*(6), 1257-1264. https://doi.org/10.5935/0103-5053.20150091
3. Alves Filho, E. G., Silva, L. M., Teofilo, E. M., Larsen, F. H., & de Brito, E. S. (2017). 1H NMR spectra dataset and solid-state NMR data of cowpea (Vigna unguiculata). *Data in Brief, 11*, 136-146. https://doi.org/10.1016/j.dib.2017.01.013.
4. Balayssac, S., Trefi, S., Gilard, V., Malet-Martino, M., Martino, R., & Delsuc, M.-A. (2009). 2D and 3D DOSY 1 H NMR, a useful tool for analysis of complex mixtures: application to herbal drugs or dietary supplements for erectile dysfunction. *Journal of pharmaceutical and biomedical analysis, 50*(4), 602-612. https://doi.org/10.1016/j.jpba.2008.10.034.
5. Davis, A. L., Cai, Y., Davies, A. P., & Lewis, J. (1996). 1H and 13C NMR assignments of some green tea polyphenols. *Magnetic Resonance in Chemistry, 34*(11), 887-890. https://doi.org/10.1002/(SICI)1097-458X(199611)34:11<887::AID-OMR995>3.0.CO;2-U.
6. Nord, L. I., Vaag, P., & Duus, J. Ø. (2004). Quantification of organic and amino acids in beer by 1H NMR spectroscopy. *Analytical chemistry, 76*(16), 4790-4798. https://doi.org/10.1021/ac0496852.
7. Wishart, D. S., Jewison, T., Guo, A. C., Wilson, M., Knox, C., Liu, Y., Djoumbou, Y., Mandal, R., Aziat, F., & Dong, E. (2012). HMDB 3.0 - the human metabolome database in 2013. *Nucleic acids research, 41*(D1), D801-D807. https://doi.org/10.1093/nar/gks1065.
8. Ye, Y., Yang, R., Lou, Y., Chen, J., Yan, X., & Tang, H. (2014). Effects of food processing on the nutrient composition of Pyropia yezoensis products revealed by NMR-based metabolomic analysis. *Journal of Food and Nutrition Research, 2*(10), 749-756. https://doi.org/10.12691/jfnr-2-10-15.
